# Construction of a Nanosensor for Non-Invasive Imaging of Hydrogen Peroxide Levels in Living Cells

**DOI:** 10.3390/biology9120430

**Published:** 2020-11-29

**Authors:** Amreen, Hayssam M. Ali, Mohammad Ahmad, Mohamed Z. M. Salem, Altaf Ahmad

**Affiliations:** 1 Department of Botany, Faculty of Life Sciences, Aligarh Muslim University, Aligarh 202002, India; gauramreen95@gmail.com (A.); aahmad.bo@amu.ac.in (A.A.); 2 Botany and Microbiology Department, College of Science, King Saud University, P.O. Box. 2455, Riyadh 11451, Saudi Arabia; hayhassan@ksu.edu.sa; 3 Department of Physics, Syracuse University, New York, NY 13244, USA; 4 Forestry and Wood Technology Department, Faculty of Agriculture (El-Shatby), Alexandria University, Alexandria 21545, Egypt; zidan_forest@yahoo.com

**Keywords:** H_2_O_2_, nanosensor, oxidative stress, fluxomics

## Abstract

**Simple Summary:**

Spatially and temporally defined H_2_O_2_ signatures are essential parts of various signaling pathways. Therefore, monitoring H_2_O_2_ dynamics with high spatio–temporal resolution is significantly important to understand how this ubiquitous signaling molecule can control diverse cellular responses. In this study, we designed and characterized a Fluorescence Resonance Energy Transfer (FRET)-based genetically encoded H_2_O_2_ sensor that provides a powerful tool to monitor the spatio–temporal dynamics of H_2_O_2_ fluxes. We have used this sensor to monitor the flux of H_2_O_2_ in live cells under stress conditions. Using this sensor, real-time information of the H_2_O_2_ level can be obtained non-invasively and would help to understand the adverse effect of H_2_O_2_ on cell physiology and its role in redox signaling.

**Abstract:**

Hydrogen peroxide (H_2_O_2_) serves fundamental regulatory functions in metabolism beyond the role as damage signal. During stress conditions, the level of H_2_O_2_ increases in the cells and causes oxidative stress, which interferes with normal cell growth in plants and animals. The H_2_O_2_ also acts as a central signaling molecule and regulates numerous pathways in living cells. To better understand the generation of H_2_O_2_ in environmental responses and its role in cellular signaling, there is a need to study the flux of H_2_O_2_ at high spatio–temporal resolution in a real-time fashion. Herein, we developed a genetically encoded Fluorescence Resonance Energy Transfer (FRET)-based nanosensor (FLIP-H_2_O_2_) by sandwiching the regulatory domain (RD) of OxyR between two fluorescent moieties, namely ECFP and mVenus. This nanosensor was pH stable, highly selective to H_2_O_2_, and showed insensitivity to other oxidants like superoxide anions, nitric oxide, and peroxynitrite. The FLIP-H_2_O_2_ demonstrated a broad dynamic range and having a binding affinity (Kd) of 247 µM. Expression of sensor protein in living bacterial, yeast, and mammalian cells showed the localization of the sensor in the cytosol. The flux of H_2_O_2_ was measured in these live cells using the FLIP-H_2_O_2_ under stress conditions or by externally providing the ligand. Time-dependent FRET-ratio changes were recorded, which correspond to the presence of H_2_O_2_. Using this sensor, real-time information of the H_2_O_2_ level can be obtained non-invasively. Thus, this nanosensor would help to understand the adverse effect of H_2_O_2_ on cell physiology and its role in redox signaling.

## 1. Introduction

Reactive oxygen species (ROS) are short-lived and highly reactive molecules formed upon incomplete reduction in oxygen [1,2]. They are the key regulators of various biological processes like signaling and development in living organisms. The higher production of ROS was observed under biotic and abiotic stress conditions [3]. The ROS can lead to oxidation of lipids, proteins, and DNA, which causes oxidative stress and finally cell death [4,5]. Recently, H_2_O_2_ has been observed as a specific component of numerous signaling pathways as well as a well-known toxic molecule among all the ROS present inside the cells [6]. Initially, the detection of ROS in cells, tissues, and organs were performed using colorimetric methods exploiting compounds such as nitroblue tetrazolium and diaminobenzidine, but these methods did not allow real-time measurements of ROS in the cells [7,8]. Later on, detection of ROS was achieved by using various fluorescent probes such as 20,70-dichlorofluorescein diacetate, dihydroethidium (DHE), dihydro-20,4,5,6,7,70-hexafluoro fluorescein but these probes are difficult to deliver into living cells and cause the toxicity [9]. Various H_2_O_2_-selective probes have been developed, which utilize a boronate-deprotection mechanism, and the detection approach depends on the selective H_2_O_2_-mediated conversion of aryl boronates to phenols [10,11]. Attachment of various fluorescent molecules with aryl boronates produces a fluorescent product when it reacts with H_2_O_2_. For investigating the changes in the endogenous level of H_2_O_2_, monoboronate-based probes have been developed Peroxyfluor-1 (PF-1), Peroxyfluor-2 (PF-2), Peroxyfluor-3 (PF-3), Peroxy Orange 1 (PO1), and Peroxy Yellow 1 (PY1). Although the H_2_O_2_ localization studies have been improved through boronate-deprotection based probes, quantitative analysis of H_2_O_2_ generation is still challenging by using these probes. Specifically, the concentration of the probe can affect the signal from the single-wavelength emitting probes. To deal with this issue, a monoboronate-based Ratio Peroxyfluor 1 (RPF)-1 probe was developed, which detects H_2_O_2_ in a ratiometric manner and can potentially permit normalization to probe concentration. However, one disadvantage with this probe is that it cannot select one kind of ROS [12].

In recent years, single fluorescent protein probes have been developed, which allowed reversible in vivo detection of reactive oxygen species in cells [9,13]. These probes include roGFP [14] and HyPer [15], which are mainly based on oxidation-reduction processes between the H_2_O_2_ and the reduced probe and show fluorescence upon oxidation. There are several disadvantages of single fluorescent protein (FP) probes, as these probes are more sensitive to some factors, i.e., pH and probe photobleaching. Therefore, to quantify the definite concentration of an analyte is not very reliable [16]. Therefore, there is a requisite to develop a simple, reliable, and non-invasive ratiometric method for the determination of H_2_O_2_ in living cells. Genetically encoded fluorescent proteins (FPs) and sensitized emission Fluorescence Resonance Energy Transfer (FRET) (seFRET)-based sensors provide an alternate way to study the metabolite dynamics in living cells. seFRET measurements are simple, and having a high Spatio–temporal resolution, enable the tracking of fast molecular events (ms). Sensing of fast changes in metabolite dynamics and protein conformational changes is possible using the sensors based on FPs [17]. These kinds of sensors have been developed for non-invasive and specific detection of metal ions, amino acids, sugars, plant hormones [18,19,20,21].

In the present work, we developed a reliable tool, FLIP-H_2_O_2_ that can measure the concentration of H_2_O_2_ in a ratiometric manner and unlike the previously reported sensors, as their measurements are affected by auto-fluorescence [14], sensor protein concentrations, and excitation wavelength in lower visible region [16]. Some of them suffer from issues, such as cell toxicity [11,12], not being ideal for long term live cell imaging and pH sensitivity [16] FP and sensing domain-based sensors have several benefits in sensitivity and specificity over sensors made from traditional materials. They operate at a similar scale to natural biological process. All these factors make FLIP-H_2_O_2_ an attractive choice for visualizing H_2_O_2_ fluxes in living cells with an extended dynamic range. The regulatory domain (RD) of *E. coli* OxyR, a transcription factor, was exploited for sensing H_2_O_2_ along with green fluorescent protein (GFP) variants for the development of a H_2_O_2_ nanosensor. In order to develop this nanosensor, cyan emitting (ECFP, donor) and yellow emitting (mVenus, acceptor) particles were genetically fused with the regulatory domain of an OxyR protein. The presence of H_2_O_2_ brought the conformational changes. This nanosensor utilizes the conformational change, and transfer of energy from the donor to acceptor fluorophore for monitoring of the H_2_O_2_. Flux of H_2_O_2_ was measured in the presence of different stress conditions in the living cells. Thus, this sensor can directly be expressed in a specific subset of cell populations and targeted to specific subcellular structures for in vivo detection and quantification of H_2_O_2_.

## 2. Materials and Methods

### 2.1. Chemicals, Vectros and Strains

All chemicals were purchased from Sigma-Aldrich (USA) unless otherwise specified. pDONR, pYES-DEST52 and pcDNA3.1(-) vectors were purchased from Thermo Fischer Scientific (USA). pGWF1 was purchased from Addgene (Watertown, MA, USA). *E. coli* strain DH5 alpha and BL21(DE3) from New England Biolabs (Beverly, MA, USA) were used for cloning and expression purpose. *Saccharomyces cerevisiae* (strain BY4742), kindly gifted by Yeast Resource Center (YRC), Washington, USA and HeLa cells purchased from ATCC (USA) were used for live cell imaging. Restriction enzymes were procured from New England Biolabs and Life technologies (USA). BP and LR clonase and other ligase enzymes were purchased from Thermo Fischer Scientific (USA). Nickel NTA (nitrilotriacetic acid) affinity chromatography columns, Imidazole, Ni^2+^- His bind resin were obtained from Novagen (USA).

### 2.2. Plasmid Construction

The regulatory domain (RD) of OxyR, a transcription factor of *Escherichia coli*, was found to sense H_2_O_2_. Therefore, it was used as the recognition element for developing the H_2_O_2_ nanosensor. The crystal structure of the protein was retrieved from RCSB-PDB (Research Collaboratory for Structural Bioinformatics-Protein Data Bank) (PDB ID–1I69, Supplementary Figure S1). The DNA sequence of the RD was extracted from the Kyoto Encyclopedia of Genes and Genomes (KEGG). ECFP and mVenus sequences were taken from addgene. ECFP and mVenus share a significant spectral overlap and Forster radius, which make this pair a good FRET pair. The nucleotide sequences of RD were amplified using *E. coli* K12 genomic DNA and gateway primers. 5′-GGG GAC AAG TTT GTA CAA AAA AGC AGG CTT CGA GAT GGC AAG CCA GCA GGG-3′ that introduces attB1 site at the 5′ end of the RD gene was used as forward primer. 5′-GGG GAC CAC TTT GTA CAA GAA AGC TGG GTC AAC CGC CTG TTT TAA AAC TTT-3′ that introduces the attB2 site was used as the reverse primer. The amplified RD nucleotide sequence was cloned in the pDONR222 vector by using gateway cloning approach (BP clonase) and the entry clone was generated (Supplementary Figure S2). We selected pGWF1, a gateway destination plasmid, as a bacterial expression vector (addgene). The property of this vector is that it already contains ECFP and mVenus fluorescent tags and gateway attachment sites. The entry clone generated during BP reaction was further used for sub-cloning in the pGWF1 vector, and results in the development of the pGWF1_ECFP_RD_mVenus construct (Supplementary Figure S3) along with in-frame 6xHis tag). The construct was checked by restriction digestion and expected bands observed on agarose gel (Supplementary Figure S4). Nucleotide sequence fidelity was confirmed by Sanger sequencing (Supplementary Figure S5). The developed FRET nanosensor for H_2_O_2_ detection was named as FLIP-H_2_O_2._ The whole construct (ECFP-RD-mVenus) was further sub-cloned in pYES-DEST52 (Supplementary Figure S6) and pcDNA3.1 (Supplementary Figure S7) for expression in *Saccharomyces cerevesie* and in mammalian cells, respectively.

### 2.3. Protein Expression and Purification

For the expression of recombinant construct, pGWF1_ECFP_RD_Venus construct was transformed to *E.coli*. The transformed cells were grown in Luria Bertini (LB) medium (HiMedia, India) containing antibiotic (ampicillin 100 µg/mL) at 37 °C up to the OD_600_ 0.6. The expression of sensor protein in bacterial cells was induced by using 1mM IPTG (Isopropyl β-D-1-thiogalactopyranoside) (Himedia, India) and grown at 21 °C for 48 h under dark conditions due to the sensitivity of fluorophores for visible light. To harvest the expressed bacterial cells, the culture was centrifuged for 10 min at 4500 rpm. Then, supernatant was discarded. Resuspension of the pellet was done in 20 mM Tris-Cl buffer (pH 8.0) with 2-mercaptoethanol (5 mM, to keep the thiol group of the RD domain in reduced form), followed by bacterial cell lysis through ultrasonication and then centrifuged at 7000 rpm for 20 min to remove the cell debris. For the purification of nanosensor protein, supernatant was syringe filtered for the removal of remaining impurities and then loaded in a Ni-NTA resin packed column and incubated at 4 °C for 4 h for the efficient binding of the recombinant His-tagged protein with resin. Washing of the column was done with buffer containing Tris-Cl (20 mM) and imidazole (20 mM). Elution buffer (Tris-Cl-20 mM; imidazole-200 mM) was used to elute the sensor protein. The purified nanosensor protein was incubated overnight at 4 °C for proper folding to its native conformation. The purity and molecular weight of nanosensor protein was checked by SDS-PAGE analysis (Supplementary Figure S8).

### 2.4. In Vitro Characterization and Ligand Binding Affinity of Nanosensor Protein

The emission spectrum of the nanosensor was monitored by using an excitation filter 430 nm/20 nm for ECFP and taking emission intensities of the nanosensor in the range of 400 to 600 nm using the spectrofluorometer (LS50B Perkin Elmer, USA). For other measurements of ECFP and mVenus emission intensities, 480 nm/20 nm and 530 nm/25 nm filters of microplate reader (Synergy H1, Biotek, USA) were used, respectively. Certain in vitro tests were also performed to check the stability and specificity of nanosensor protein in different buffer systems PBS (Phosphate buffer saline), MOPS (3-(N-morpholino) propanesulfonic acid), Tris-Cl, and TBS (Tris-buffered saline). Furthermore, the stability of nanosensor protein was also checked in pH from 5.0–8.0 in the presence of H_2_O_2_ (1 µM) and the absence of H_2_O_2_. The PBS buffer at pH 7.0 was found to be suitable and selected for further analysis. The FRET ratio was recorded after adding the 20 µL of ligand with 180 µL of the diluted protein sample. The ligand titration curve was obtained by titrating purified protein with different concentration of H_2_O_2,_ ranging from nanomolar to millimolar. The affinity of nanosensor protein (*K*_d_) was determined by using the change in mVenus/ECFP ratio (FRET ratio) after binding with H_2_O_2_ by applying the ligand titration curves to the equation: S = (r − r_apo_)/(r_sat_ − r_apo_) = [H_2_O_2_]/(*K*_d_ + [H_2_O_2_]), where S is saturation; r is ratio; r_apo_ is ratio in the absence of H_2_O_2_; r_sat_ is ratio at saturation with H_2_O_2_.

### 2.5. In Vivo Characterization in Bacterial Cells

The pGWF1_ECFP_RD_Venus transformed bacterial cells (*E.coli* BL21) were grown in LB medium at 37 °C for in vivo analysis of the nanosensor. Expression of the nanosensor protein was induced by adding 1 mM IPTG (Isopropyl β-D-1-thiogalactopyranoside) to the cultured bacterial cells, and then further culture for 48 h at 21 °C. The bacterial cells were harvested through centrifugation, and the pellet was dissolved in PBS buffer (pH 6.5). Various level of salt treatment (0, 100 mM and 200 mM NaCl) and arsenic (sodium arsenate) treatment (10 µM) were given to produce H_2_O_2_ inside the bacterial cells. The 180 µL bacterial cells expressing nanosensor protein was transferred to 96-well plate and the FRET ratio was recorded for 420 s at regular interval of 60 s under normal conditions after the addition of 20 µL of different concentrations of H_2_O_2_ (0 mM, 0.5 mM and 1 mM). FRET ratio was also recorded under stress conditions (with arsenic and NaCl). Initially, fluorescence emission of acceptor and donor was measured for baseline correction and then measurement of the FRET ratio was done after 2 min of the addition of 10 µM arsenic (sodium arsenate) and 100 mM and 200 mM NaCl. The measurements were taken for further 18 min at every 2 min internals.

### 2.6. H_2_O_2_ Dynamics in Yeast under Normal and Stress Conditions

Live cell imaging of FLIP-H_2_O_2_ expressing yeast cells, and dynamics of H_2_O_2_ in the yeast cells under stress conditions using FLIP-H_2_O_2_ were carried out in the presence of arsenic and salt treatment (200 mM) for 10 min as per the protocol of Mohsin and Ahmad [19]. *Saccharomyces cerevesie* strain BY4742 was transformed with gateway cloned pYES-DEST-ECFP_RD_mVenus. The dual emission intensity ratio (mVenus(Y)/ECFP(C) ratio) was monitored by using confocal microscope (SP5, Leica Microsystems, Wetzlar, Germany) which is controlled by LAS-AF (Leica Application Suite-Advanced Fluoresence) software (Leica, Germany).

### 2.7. Monitoring of H_2_O_2_ Changes in Mammalian Cells

Live cell imaging and measurement of the change in the level of the H_2_O_2_ in the mammalian cells (HeLa cells) expressing FLIP-H_2_O_2_ was carried out as per Ahmad et al. [22].

## 3. Results and Discussion

### 3.1. Designing, Construction and Spectral Analysis of FLIP-H_2_O_2_

**Figure 1:**
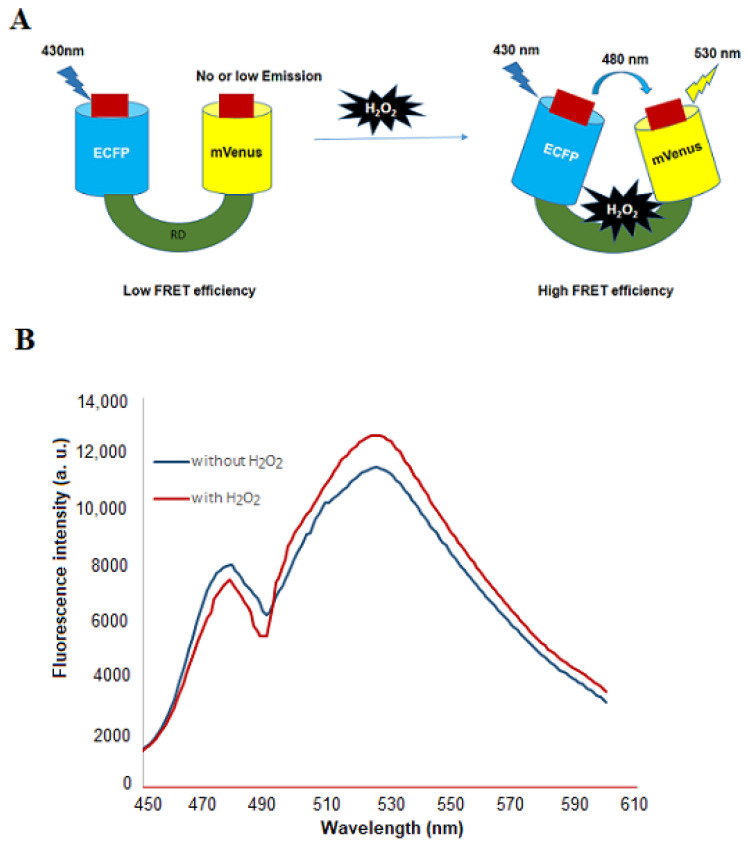
Schematic representation of the FLIP-H_2_O_2_ nanosensor. (**A**) Regulatory domain is attached with donor (ECFP) and acceptor (mVenus). Binding of H_2_O_2_ with the RD brings the donor and acceptor in close proximity and at this stage, emission of ECFP results in the transfer of energy and excites the mVenus. Ratio changes occur in the mVenus/ECFP emission by H_2_O_2_. (**B**) Spectral analysis was performed using purified sensor protein.

In this study, a genetically encoded nanosensor was developed for non-invasive real-time measurement of H_2_O_2_ in living cells by using bright fluorophores and advanced fluorescence imaging. The regulatory domain (RD) of OxyR, a member of the LysR family of bacterial transcription factor, was used as a ligand sensory domain (Supplementary Figure S1). Previous studies reported that the regulatory domain binds to H_2_O_2_ and an intramolecular disulphide bond is formed between Cys-199 and Cys-208 due to which structural changes occur in the regulatory domain that fetches both termini close to each other [23]. The RD of OxyR in reduced and oxidized form was shown in supplemental Figure S9. Significant conformational changes occurred in RD due to the disulphide bond formation between specific cysteines residues (Supplementary Figure S10). The two variants of green fluorescent protein were ligated with the H_2_O_2_ sensory domain, RD, at the N and C-terminus, respectively. For the development of these genetically encoded FRET sensors, the prerequisite is that the ligand sensory domain must be undergoing the appropriate conformational changes, which is translated the ligand binding into the FRET [24]. The sensing element, RD, fulfils this criterion of FRET as there was significant variation in the emission intensity of acceptor and donor fluorophore with the addition of the H_2_O_2_. The schematic mechanism of FLIP-H_2_O_2_ sensor is represented in Figure 1A. Spectral analysis was done for the validation of expressed nanosensor protein. The nanosensor protein showed changes in the fluorescence emission intensities of ECFP and mVenus in the presence of H_2_O_2_. Addition of H_2_O_2_ decreases the emission intensity of ECFP and increases the emission intensity of mVenus confirming that FRET is occurring in the presence of H_2_O_2_ with the nanosensor protein (Figure 1B).

### 3.2. In Vitro Characterization of FLIP-H_2_O_2_ Nanosensor

**Figure 2:**
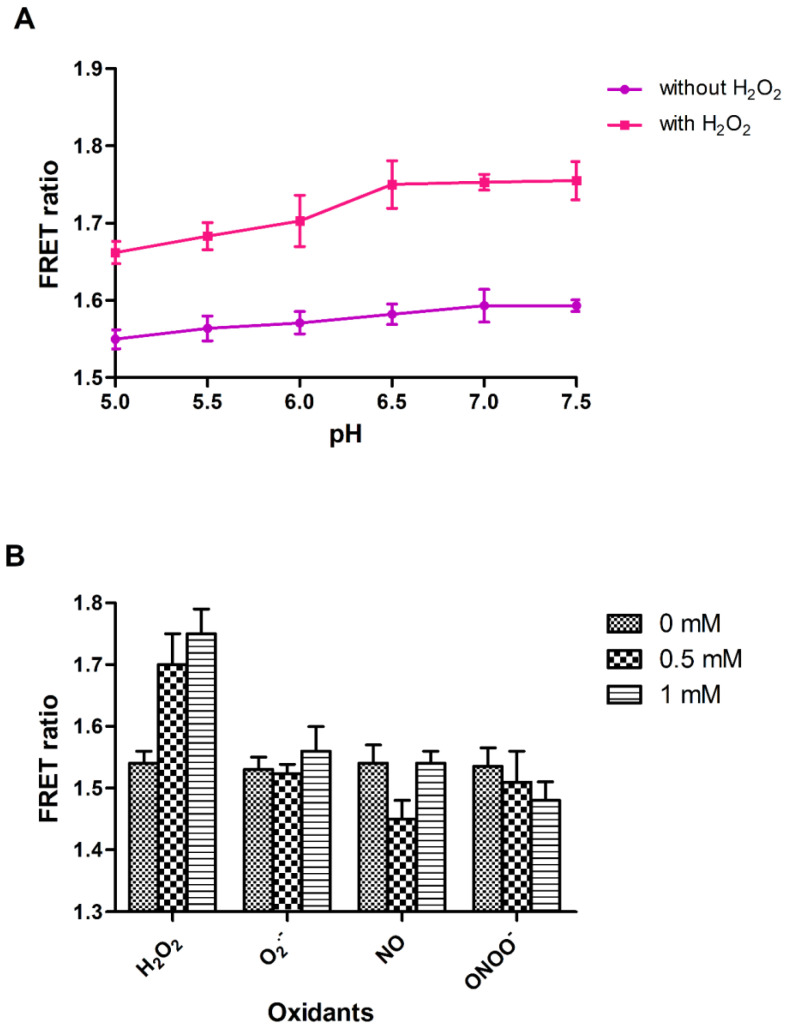
In vitro analysis of FLIP-H_2_O_2_. (**A**) pH stability analysis of FLIP-H_2_O_2_. (**B**) Specificity analysis of the FLIP-H_2_O_2_ nanosensor. (H_2_O_2_—hydrogen peroxide, O_2_^−^—superoxide anions, NO—nitric oxide, ONOO^−^—peroxynitrite). Data are mean of three independent experiments. Vertical bars represent the standard error.

Fluorescent proteins are pH-sensitive, and their stability depends on the pH and composition of the buffer. To test the pH sensitivity and stability of nanosensor, fluorescence measurements were performed in different buffers (PBS, MOPS, Tris-Cl, and TBS) and at various pH ranges (5.0–8.0). The nanosensor protein exhibit the optimum stability in the PBS buffer (pH 7.0), as with this buffer the least changes in the FRET ratio were observed (Figure 2A). In the absence of H_2_O_2_, the purified protein showed no change in FRET ratio. However, after the addition of H_2_O_2_ to the purified sensor protein, which is suspended in PBS buffer, the variation in FRET ratio was found (Figure 2A). Therefore, PBS buffer (pH 7.0) was selected to carry out further investigation of nanosensor. Specificity of the nanosensor was measured in vitro with various oxidants. The addition of oxidants such as super-oxide anion, nitric oxide and peroxinitrite did not cause any significant change in the FRET ratio of sensor protein (Figure 2B).

**Figure 3:**
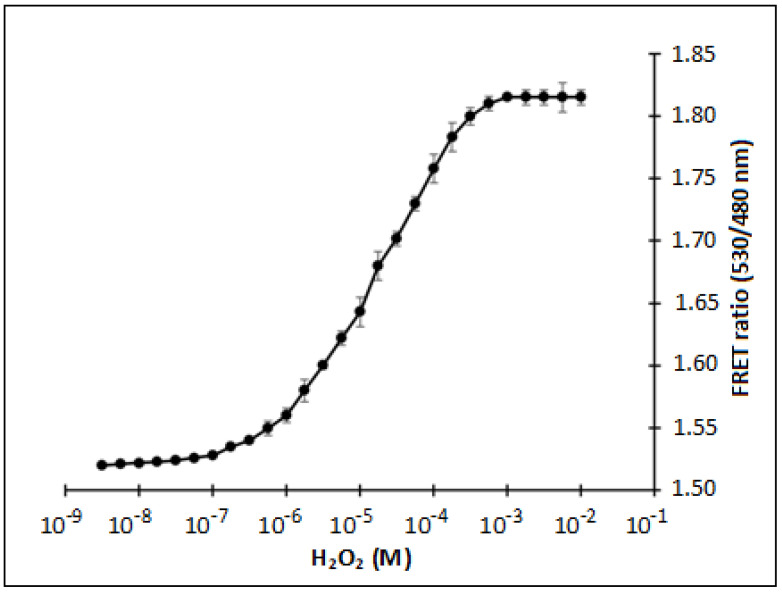
In vitro titration analysis of FLIP-H_2_O_2_ with various concentration of H_2_O_2,_ the sigmoidal graph representing the changes in the Fluorescence Resonance Energy Transfer (FRET) ratio at very low to high concentration of H_2_O_2_ and saturation of FLIP-H_2_O_2_ nanosensor. Data are mean of three independent experiments. Vertical bars represent the standard error.

To find out the saturation limit and K_d_ of nanosensor protein, a titration experiment was conducted with nanosensor protein by adding the H_2_O_2_ from nanomolar to millimolar series (Figure 3). The calculated affinity (K_d_) of sensor with the H_2_O_2_ was found to be 247 µM. FRET ratio measurement approach based on donor and acceptor fluorophore emission intensities has previously been used for the monitoring of antioxidants and amino acids [25,26]. In case of Hyper, authors showed sub-micro molar affinity for H_2_O_2_ in bacterial cells and higher affinity in HeLa cells and reported that in living cells at least 5 µM H_2_O_2_ is required to induce the fluorescence changes [15]. Exact *K*_d_ value was not mentioned in this case, however we expect this value to be around 8 µM. *K*_d_ reported in the literature for these systems is comparable with *K*_d_ value reported in our case and it is in this range. Limit of detection reported in case of the other H_2_O_2_ sensor was in the range of 1 to 200 µM [12,27,28]. FLIP-H_2_O_2_ showed a detection range from sub-micromolar to micromolar range (~0.1 to 563 µM). Sensor based on FRET offers higher dynamic range in terms of detection. For superior performance at nanomolar level, its limit of detection can further be improved in future. A comparative analysis of major H_2_O_2_ detection method has been presented in Supplementary Table S1.

### 3.3. Intracellular H_2_O_2_ Flux Monitoring in Bacterial Cells

**Figure 4:**
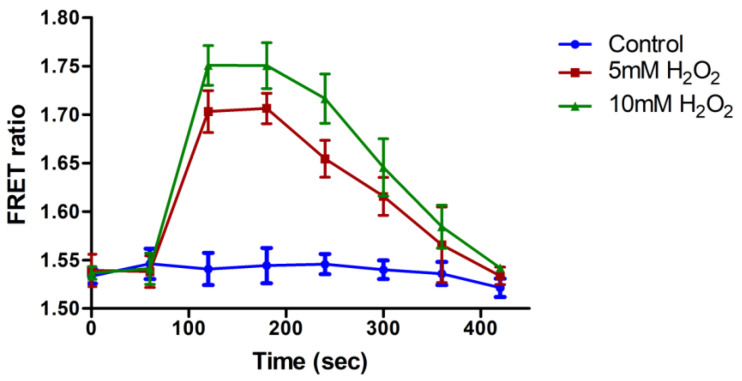
In vivo H_2_O_2_ flux analysis using FLIP-H_2_O_2_. Nanosensor expressing bacterial cells were incubated with various concentration of H_2_O_2_ and FRET ratio was monitored for 420 s. Data are the mean of three independent experiments. Vertical bars represent the standard error.

For in vivo H_2_O_2_ flux analysis, the *E. coli* BL21 cells were transformed with pGWF1-ECFP_RD_mVenus and allowed to express in the cytosol. The harvested bacterial cells were resuspended in PBS buffer and then resuspended bacterial cells were transferred to microplate wells and supplemented with H_2_O_2_. After a fixed interval, the emission intensities of both fluorophores were recorded using fluorescence plate reader. An increase in the FRET ratio from 1.53 to 1.70 and 1.53 to 1.75 after the addition of 0.5 mM and 1 mM of H_2_O_2,_ respectively (Figure 4). Previously, the measurement of metabolite and ion accumulation was also performed inside the bacterial cells using FRET-based nanosensors [26,29].

### 3.4. Monitoring of H_2_O_2_ in Yeast and Mammalian Cells

**Figure 5:**
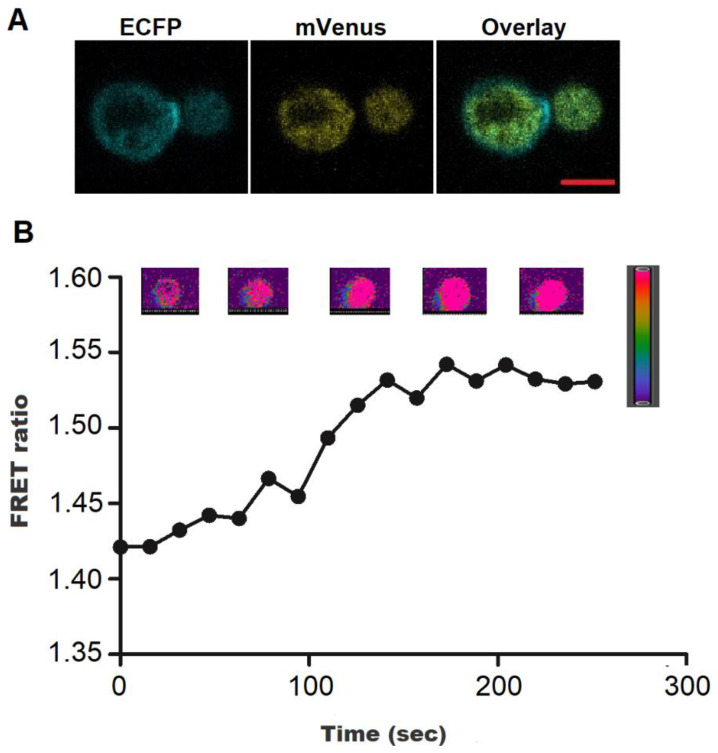
Flux monitoring of H_2_O_2_ in the cytosol of yeast cells. (**A**) Confocal images of yeast cells expressing the FLIP-H_2_O_2_, showing ECFP, mVenus and Overlay (Scale bar = 2 μm). (**B**) H_2_O_2_ was added externally to the yeast cells after 1 min of laser scan and ratiometric images were recorded at defined intervals. Data are the mean of three independent experiments. Vertical bars represent the standard error.

Real-time confocal imaging data obtained from sensor transformed yeast cells indicated that the FLIP-H_2_O_2_ was expressed in the cytosol (Figure 5A) and allowed monitoring of H_2_O_2_ inside the cells. After one minute of addition of H_2_O_2_, FLIP-H_2_O_2_ expressing yeast cells showed an increase in the FRET ratio from 1.42 to 1.55 (Figure 5B). Live cell imaging was also performed earlier for the monitoring of 2-oxoglutarate and ajmalacine in yeast cells using FRET-based 2-oxoglutarate and ajmalacine nanosensors [30,31].

**Figure 6:**
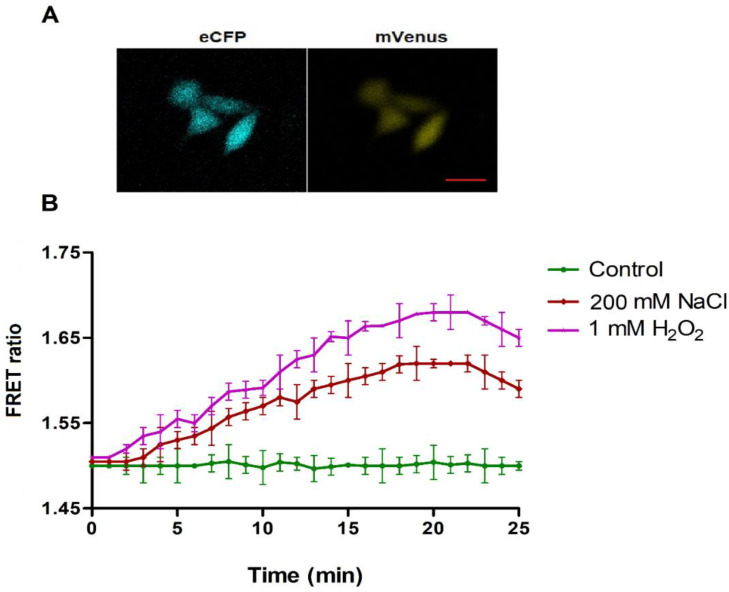
Flux monitoring of H_2_O_2_ in mammalian cells. (**A**) Confocal imaging of FLIP-H_2_O_2_ transformed mammalian cells (Scale bar = 10 μm). (**B**) Time dependent acceptor/donor intensity ratio change in single HeLa cells in the presence of externally supplied H_2_O_2_ and NaCl. Data are mean of three independent experiments. Vertical bars represent the standard error.

In the case of yeast, nanosensor showed a kind of fluorescence quenching which was reflected in the form of initial low fluorescence intensity. Nanosensor had reacted to the H_2_O_2_ fast, so it reached to the saturation quickly after addition in these cells. Confocal imaging of transiently transfected HeLa cells with FLIP-H_2_O_2_ showed that FLIP-H_2_O_2_ distributed predominantly in the cytoplasm of cultured cells (Figure 6A). Non-invasive analysis of transfected HeLa cells showed an increase in the FRET ratio from 1.51 to 1.66 and 1.50 to 1.62 by the addition of H_2_O_2_ and NaCl respectively (Figure 6B). NaCl induced H_2_O_2_ production was also reported in endothelial cells where it was observed that the extracellular volume expands due to increased salt intake and induces the activation of transforming growth factor-β (TGF-β) [32]. However, TGF-β increases endothelial NADPH (nicotinamide adenine dinulceotide phosphate) oxidase-4 [33], an enzyme that induces the production of H_2_O_2_ [34].

### 3.5. In Vivo H_2_O_2_ Flux Monitoring under Stress Conditions

**Figure 7:**
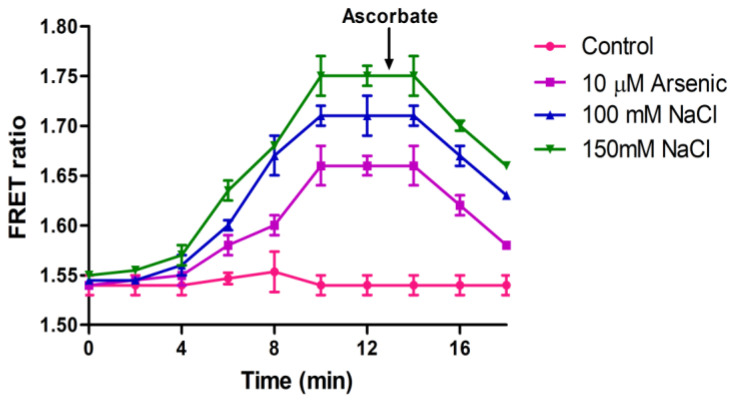
Change in the FRET ratio in the presence of different concentration of salt and arsenic in the bacterial cells expressing FLIP-H_2_O_2_. Data are mean of three independent experiments. Vertical bars represent the standard error.

**Figure 8:**
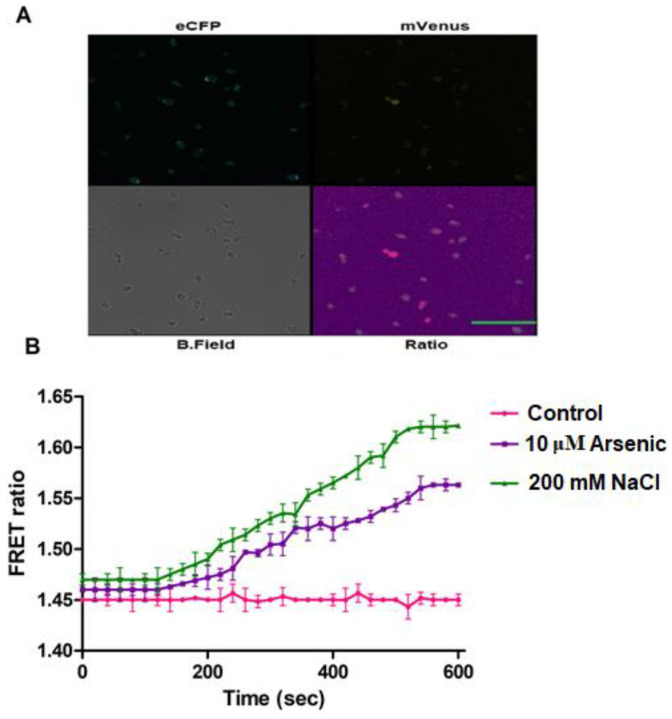
(**A**) Confocal microscopy imaging of nanosensor expressing yeast cells showing ECFP, mVenus, bright field and ratio images at various channels (Scale bar = 20 μm.). (**B**) Ratiometric measurements of H_2_O_2_ was performed in yeast cells after one minute of addition of As^3+^ and NaCl. Data are the mean of three independent experiments. Vertical bars represent the standard error.

To analyze and monitor the effect of abiotic stress conditions on the generation of H_2_O_2_, NaCl and arsenic were provided externally and fluorescence intensity changes were monitored in a ratiometric manner in FLIP-H_2_O_2_ expressing yeast and bacterial cells using microplate reader. For the generation of abiotic stress, we treated bacterial and yeast cells with different concentrations of NaCl and arsenic, separately. FRET ratio was monitored at the interval of 2 min for 18 min. In the first 2 min, no significant change in the FRET ratio was observed because the expression of stress producing enzymes and synthesis of H_2_O_2_ in bacterial and yeast cells takes time. In arsenic and salt treated cells, an increase in the FRET ratio was observed after 2 min, which was increased up to 10 min and then become saturated (Figure 7 and Figure 8A,B). At the saturation point, ascorbate was added to check and measure the effect of this antioxidant on cellular H_2_O_2_. Nanosensor reports the decreased level of H_2_O_2_ inside the bacterial cells in the presence of ascorbate (Figure 7), which indicate its antioxidant property. Experimental results have indicated the generation of H_2_O_2_ after exposure to arsenic in Chinese hamster ovary cells [35].

## 4. Conclusions

Understanding how living cells cope with environmental change requires a Spatio–temporal perspective. The development and adaptation of a FRET nanosensor provides the capacity to visualize changes in the transport and accumulation of metabolites and small molecules at the cellular level, which is required to identify the regulatory switches in the metabolic network. Flux monitoring of H_2_O_2_ in the cytosol of living bacterial, yeast and mammalian cells demonstrate that the FLIP-H_2_O_2_ is an excellent tool for non-invasive real-time monitoring of H_2_O_2_ in any cell type. Moreover, attachment of sub-cellular targeting signal to the FLIP-H_2_O_2_ will facilitate in vivo monitoring of H_2_O_2_ in key cellular organelles, such as chloroplasts, mitochondria. Therefore, live imaging approaches coupled with automated image-analysis algorithms are revealing new levels of dynamism and plasticity of the cellular response. Together, these tools will offer a more comprehensive understanding of environmental responses in cellular organisms.

## Supplementary Materials

The following are available online at https://www.mdpi.com/2079-7737/9/12/430/s1, Figure S1: Crystal structure of regulatory domain of OxyR which was used as sensory element, Figure S2, Schematic representation of pDONR-RD, Figure S3, Representation of full construct of pGWF1-ECFP-RD-mVenus, Figure S4, Restriction digestion of pGWF1-ECFP-RD-mVenus plasmid. Digested product was resolved on 1% agarose gel and visualized by EtBr/UV, Figure S5, Nucleotides sequences of the FLIP-H_2_O_2_ sensor, Figure S6, Schematic representation of pYES-DEST-ECFP-RD-mVenus, Figure S7, Schematic representation of pcDNA-ECFP-RD-mVenus, Figure S8, Purified ECFP-RD-mVenus protein was resolved on 12% SDS-PAGE. Expected band was observed (M-marker, S-purified protein ~80 kD), Figure S9, Reduced and oxidized form of RD and H_2_O_2_ induced conformational changes in the domain, Figure S10, Close-up structure of critical cysteine residues of Regulatory Domain involved in H_2_O_2_ sensing, Table S1, Comparison of various H2O2 detection approaches and their properties.
