# Supplementary material for: Construction of a Nanosensor for Non-Invasive Imaging of Hydrogen Peroxide Levels in Living Cells
Source: Biology (Basel). 2020 Nov 29;9(12):430. doi: 10.3390/biology9120430 (PMC7760702; doi:10.3390/biology9120430)
Supplement: Supplementary file 1 [file biology-09-00430-s001.pdf]

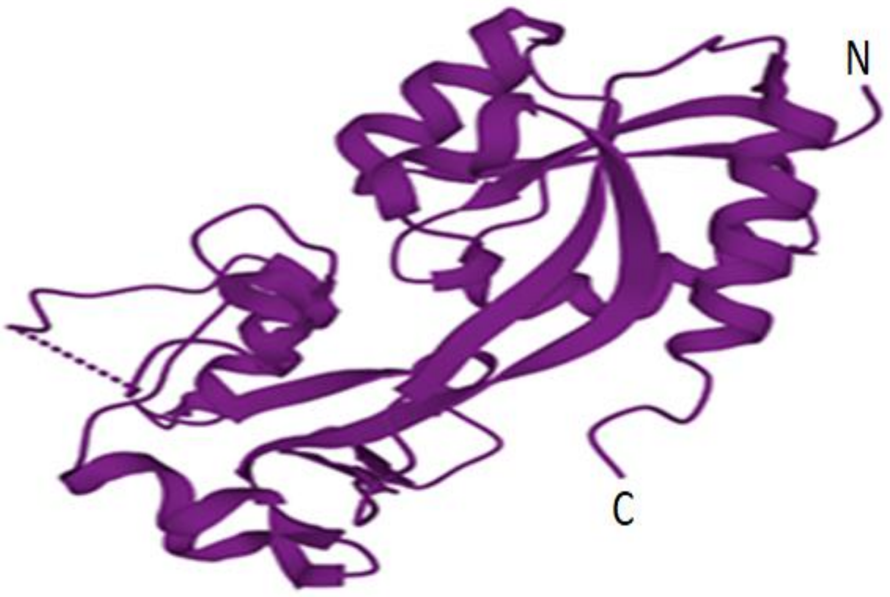

Fig.S1 Crystal structure of regulatory domain of OxyR which was used as sensory element.

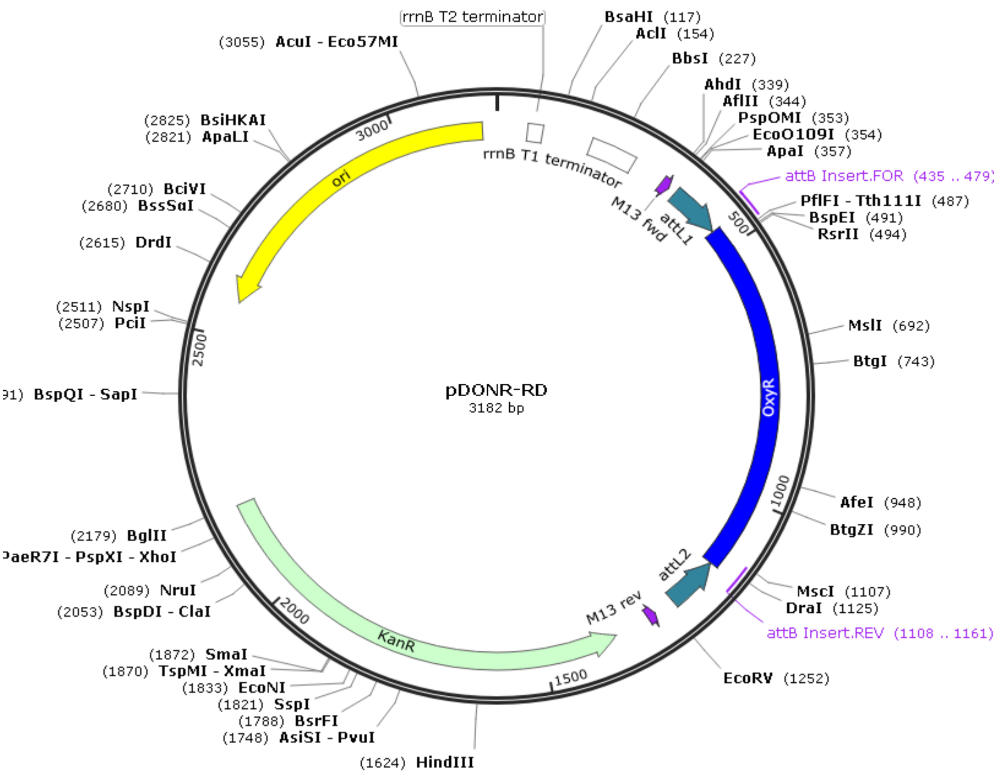

Fig. S2 Schematic representation of pDONR-RD

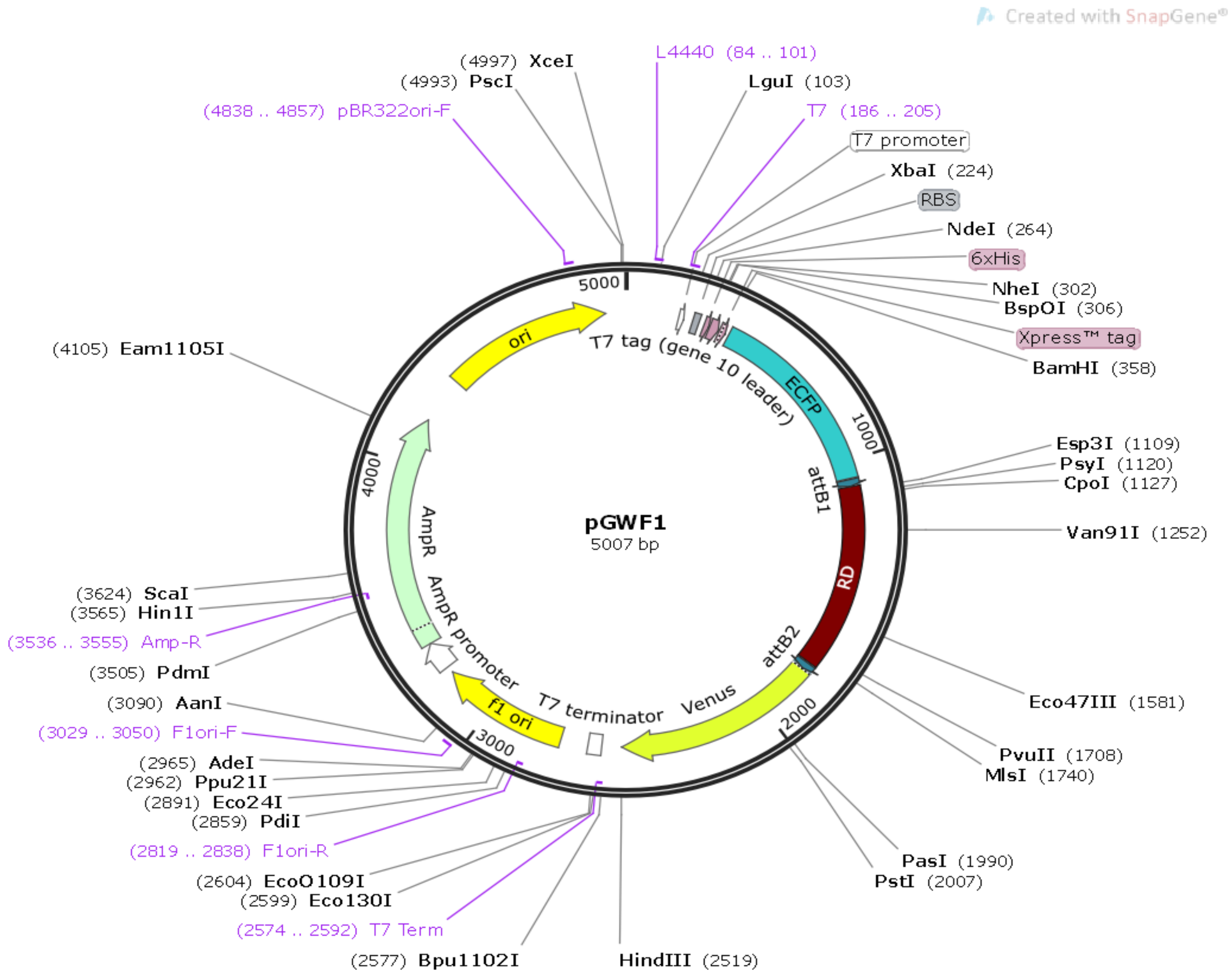

.

Fig. S3 Representation of full construct of pGWF1-ECFP-RD-mVenus

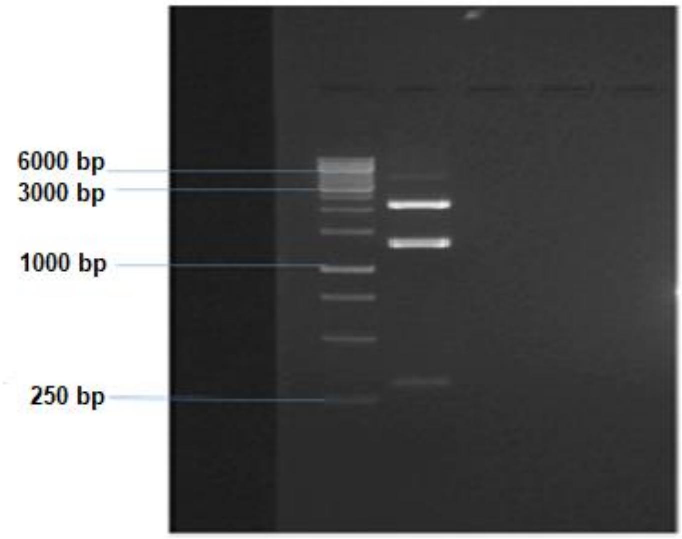

Fig. S4 Restriction digestion of pGWF1-ECFP-RD-mVenus plasmid. Digested product was resolved on 1% agarose gel and visualized by EtBr/UV.

5’ATGGTGAGCAAGGGCGAGGAGCTGTTCACCGGGGTGGTGCCCATCCTGGTCGAGCTGGACGGCGACGTA
AACGGCCACAAGTTCAGCGTGTCCGGCGAGGGCGAGGGCGATGCCACCTACGGCAAGCTGACCCTGAAGTT
CATCTGCACCACCGGCAAGCTGCCCGTGCCCTGGCCCACCCTCGTGACCACCCTGACCTGGGGCGTGCAGT
GCTTCAGCCGCTACCCCGACCACATGAAGCAGCACGACTTCTTCAAGTCCGCCATGCCCGAAGGCTACGTCC
AGGAGCGCACCATCTTCTTCAAGGACGACGGCAACTACAAGACCCGCGCCGAGGTGAAGTTCGAGGGCGAC
ACCCTGGTGAACCGCATCGAGCTGAAGGGCATCGACTTCAAGGAGGACGGCAACATCCTGGGGCACAAGCT
GGAGTACAACTACATCAGCCACAACGTCTATATCACCGCCGACAAGCAGAAGAACGGCATCAAGGCCAACTT
CAAGATCCGCCACAACATCGAGGACGGCAGCGTGCAGCTCGCCGACCACTACCAGCAGAACACCCCCATCG
GCGACGGCCCCGTGCTGCTGCCCGACAACCACTACCTGAGCACCCAGTCCGCCCTGAGCAAAGACCCCAAC
GAGAAGCGCGATCACATGGTCCTGCTGGAGTTCGTGACCGCCGCCGGGATCACTGGGGACAAGTTTGTACA
AAAAAGCAGGCTTGAGATGGCAAGCCAGCAGGGCGAGACGATGTCCGGACCGCTGCACATTGGTTTGATTC
CCACAGTTGGACCGTACCTGCTACCGCATATTATCCCTATGCTGCACCAGACCTTTCCAAAGCTGGAAATGT
ATCTGCATGAAGCACAGACCCACCAGTTACTGGCGCAACTGGACAGCGGCAAACTCGATTGCGTGATCCTC
GCGCTGGTGAAAGAGAGCGAAGCATTCATTGAAGTGCCGTTGTTTGATGAGCCAATGTTGCTGGCTATCTAT
GAAGATCACCCGTGGGCGAACCGCGAATGCGTACCGATGGCCGATCTGGCAGGGGAAAAACTGCTGATGCT
GGAAGATGGTCACTGTTTGCGCGATCAGGCAATGGGTTTCTGTTTTGAAGCCGGGGCGGATGAAGATACAC
ACTTCCGCGCGACCAGCCTGGAAACTCTGCGCAACATGGTGGCGGCAGGTAGCGGGATCACTTTACTGCCA
GCGCTGGCTGTGCCGCCGGAGCGCAAACGCGATGGGGTTGTTTATCTGCCGTGCATTAAGCCGGAACCACG
CCGCACTATTGGCCTGGTTTATCGTCCTGGCTCACCGCTGCGCAGCCGCTATGAGCAGCTGGCAGAGGCCA
TCCGCGCAAGAATGGATGGCCATTTCGATAAAGTTTTAAAACAGGCGGTTGGGGACCACTTTGTACAAGAAA
GCTGGGTCGTGAGCAAGGGCGAGGAGCTGTTCACCGGGGTGGTGCCCATCCTGGTCGAGCTGGACGGCGA
CGTAAACGGCCACAAGTTCAGCGTGTCCGGCGAGGGCGAGGGCGATGCCACCTACGGCAAGCTGACCCTGA
AGCTGATCTGCACCACCGGCAAGCTGCCCGTGCCCTGGCCCACCCTCGTGACCACCCTGGGCTACGGCCTG
CAGTGCTTCGCCCGCTACCCCGACCACATGAAGCAGCACGACTTCTTCAAGTCCGCCATGCCCGAAGGCTAC
GTCCAGGAGCGCACCATCTTCTTCAAGGACGACGGCAACTACAAGACCCGCGCCGAGGTGAAGTTCGAGGG
CGACACCCTGGTGAACCGCATCGAGCTGAAGGGCATCGACTTCAAGGAGGACGGCAACATCCTGGGGCACA
AGCTGGAGTACAACTACAACAGCCACAACGTCTATATCACCGCCGACAAGCAGAAGAACGGCATCAAGGCC
AACTTCAAGATCCGCCACAACATCGAGGACGGCGGCGTGCAGCTCGCCGACCACTACCAGCAGAACACCCC
CATCGGCGACGGCCCCGTGCTGCTGCCCGACAACCACTACCTGAGCTACCAGTCCGCCCTGAGCAAAGACC
CCAACGAGAAGCGCGATCACATGGTCCTGCTGGAGTTCGTGACCGCCGCCGGGATCACTCTCGGCATGGAC
GAGCTGTACAAGTAAA3’

Fig. S5 Nucleotides sequences of the FLIP-H_2O_2 sensor

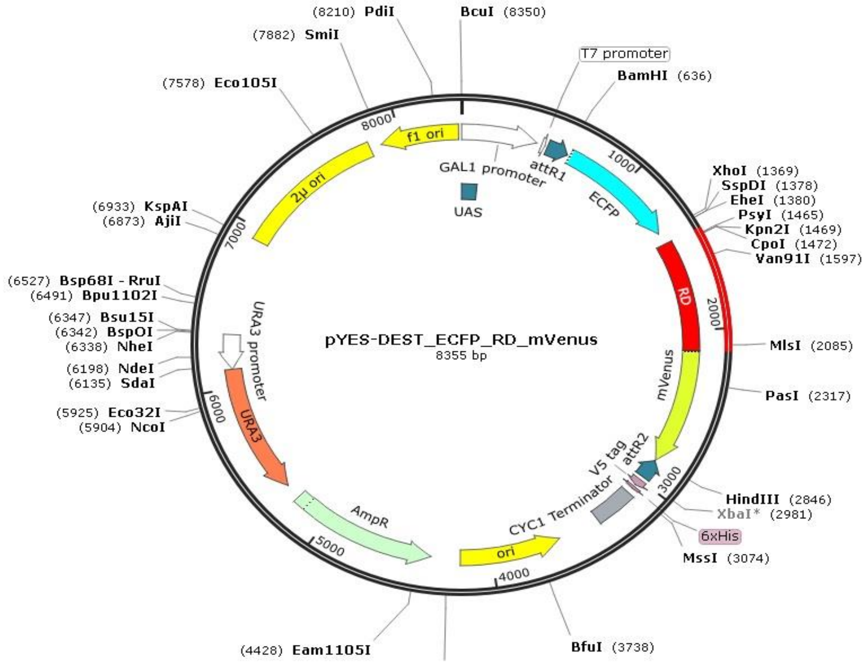

Fig. S6 Schematic representation of pYES-DEST-ECFP-RD-mVenus

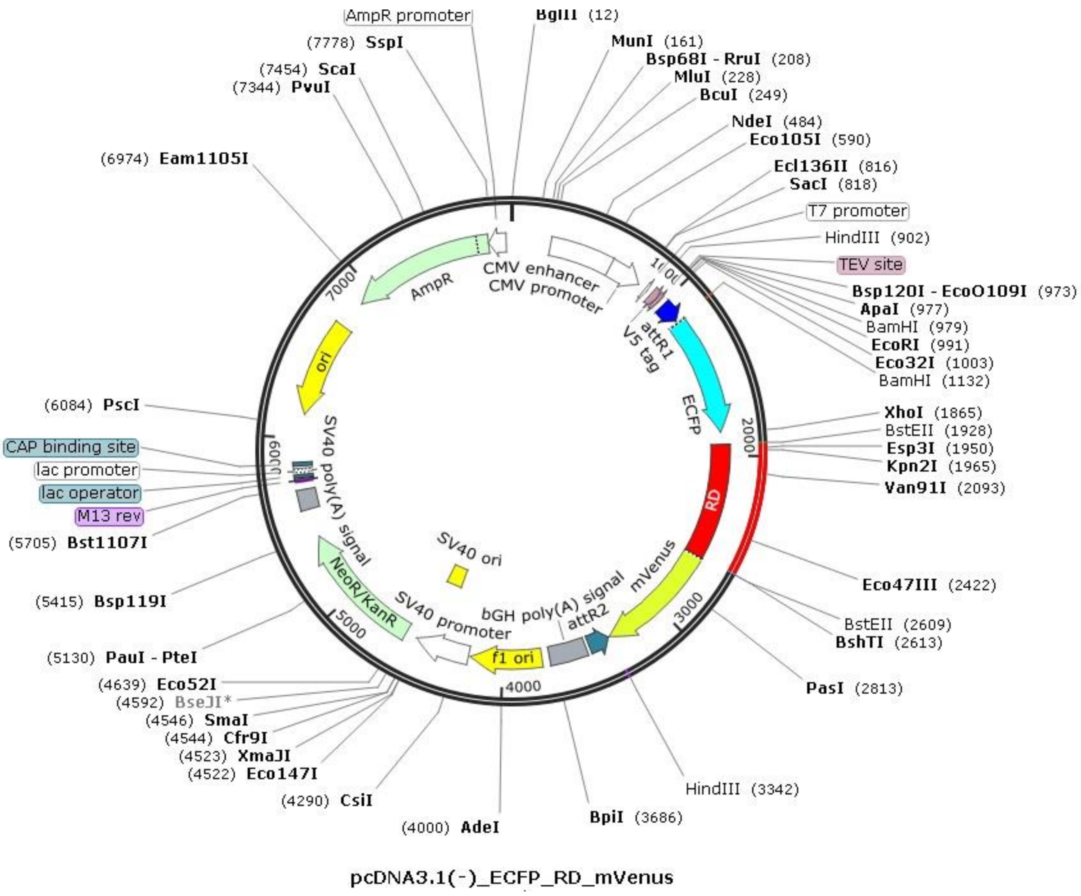

Fig. S7 Schematic representation of pcDNA 3.1(-)-ECFP-RD-mVenus

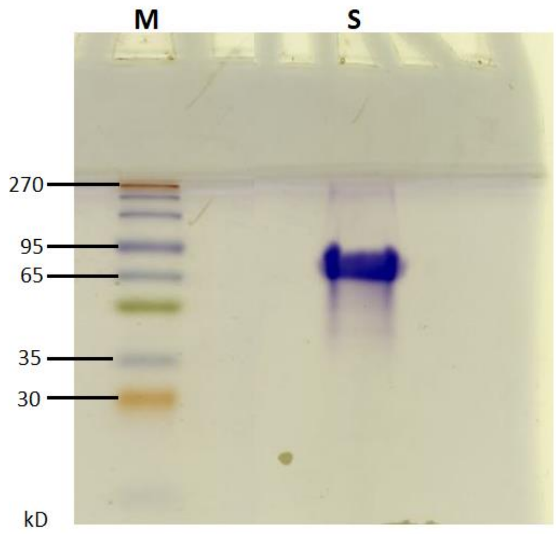

Fig. S8 Purified ECFP-RD-mVenus protein was resolved on 12% SDS-PAGE. Expected band was observed (M-marker, S-purified protein ~80 kD)

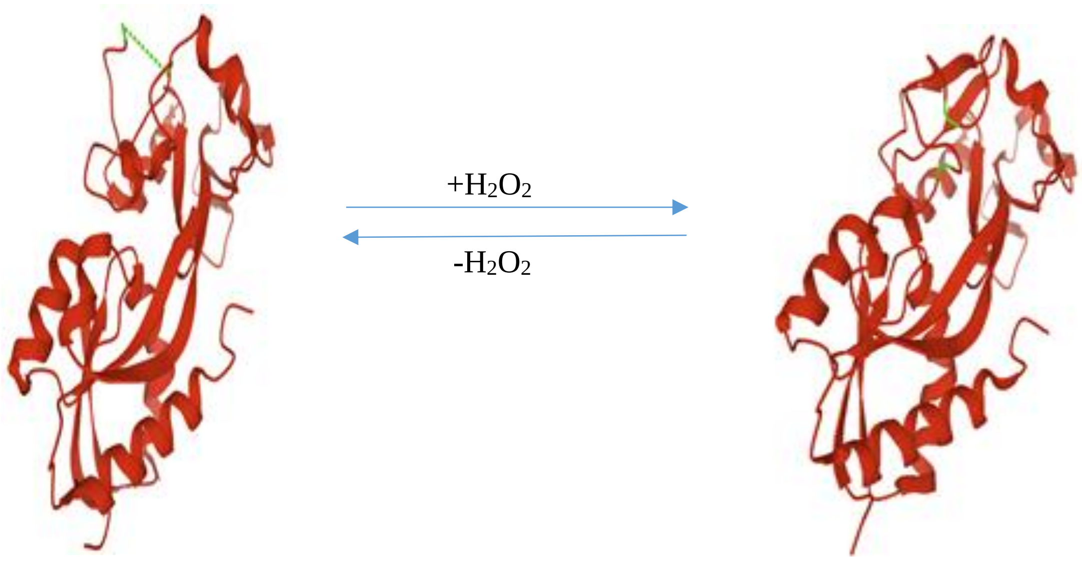

Fig. S9 Reduced and oxidized form of RD and H_2O_2 induced conformational changes in the domain.

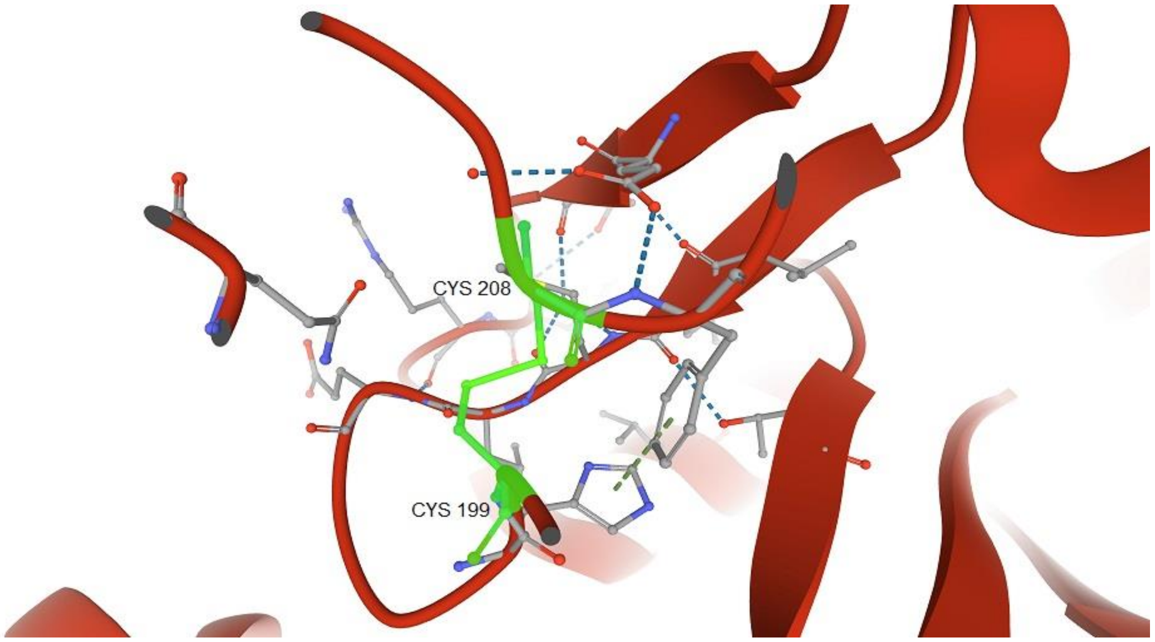

Fig. S10 Close-up structure of critical cysteine residues of regulatory domain involved in H_2O_2 sensing.

Supplementary table. S1 Comparison of various H_2O_2 detection approaches and their properties.

**Sensor/ Probe name**	**Advantages**	**Disadvantages**	**Kd**	**Linear detection range** (μM)	**References**
Luminol Chemiluminescent Probe	High signal-to background ratio, Spectral properties can be easily adjusted.	Temperature dependency, Irreversible, Reaction dependent interferences (e.g., Ag(I), Cu(II), Co(II), Fe(II), pH, ROS, hydroperoxides).	Not reported	1-100	[27]
RPF1	Ratiometric fluorescence reporter, minimize damage and autofluorescence from biological samples	difficult to deliver into living cells and causes the toxicity, not rapid response to peroxide	Not reported	5-200	[12]
Dichlorodihydrofluorescein diacetate (DCFH-DA)	cell-permeable	DCFH does not directly react with H_2O_2, can actually produce $O_2^{\bullet-}$	Not reported	1-50	[9]
roGFP	Dynamic changes can be monitored, Reversible reaction	Single fluorophore based Approach, low dynamic rang prone to photobleaching.	Not defined	10-1000	[28]
Hyper	Dynamic changes can be monitored, Reversible reaction, Can be targeted to specific subcellular compartments	Highly sensitive to pH changes	Sub-micromolar	0.2-10	[15]

.
